# Lipopolysaccharide Induces Endoplasmic Store Ca^2+^-Dependent Inflammatory Responses in Lung Microvessels

**DOI:** 10.1371/journal.pone.0063465

**Published:** 2013-05-10

**Authors:** Kathirvel Kandasamy, Lavanya Bezavada, Rachel B. Escue, Kaushik Parthasarathi

**Affiliations:** 1 Department of Physiology, The University of Tennessee Health Science Center, Memphis, Tennessee, United States of America; 2 Department of Orthopedic Surgery and Biomedical Engineering, The University of Tennessee Health Science Center, Memphis, Tennessee, United States of America; Duke University Medical Center, United States of America

## Abstract

The pulmonary microvasculature plays a critical role in endotoxin-induced acute lung injury. However, the relevant signaling remain unclear. Specifically the role of endothelial Ca^2+^ in the induction of endotoxin-mediated responses in lung microvessels remains undefined. Toward elucidating this, we used the isolated blood-perfused rat lung preparation. We loaded microvessels with the Ca^2+^ indicator, Fura 2 AM and then determined Ca^2+^ responses to infusions of lipopolysaccharide (LPS) into the microvessels. LPS induced a more than two-fold increase in the amplitude of cytosolic Ca^2+^ oscillations. Inhibiting inositol 1,4,5 trisphosphate receptors on endoplasmic reticulum (ER) Ca^2+^ stores with Xestospongin C (XeC), blocked the LPS-induced increase in the Ca^2+^ oscillation amplitude. However, XeC did not affect entry of external Ca^2+^ via plasma membrane Ca^2+^ channels in lung microvascular endothelial cells. This suggested that LPS augmented the oscillations via release of Ca^2+^ from ER stores. In addition, XeC also blocked LPS-mediated activation and nuclear translocation of nuclear factor-kappa B in lung microvessels. Further, inhibiting ER Ca^2+^ release blunted increases in intercellular adhesion molecule-1 expression and retention of naïve leukocytes in LPS-treated microvessels. Taken together, the data suggest that LPS-mediated Ca^2+^ release from ER stores underlies nuclear factor-kappa B activation and downstream inflammatory signaling in lung microvessels. Thus, we show for the first time a role for inositol 1,4,5 trisphosphate-mediated ER Ca^2+^ release in the induction of LPS responses in pulmonary microvascular endothelium. Mechanisms that blunt this signaling may mitigate endotoxin-induced morbidity.

## Introduction

Sepsis is characterized by rapid retention of leukocytes in lung microvessels. Vascular administration of endotoxin (lipopolysaccharide; LPS), a model of sepsis, reveals a major role for the endothelium in this process. Using real-time fluorescence imaging, we recently reported that targeted infusion of LPS into lung capillaries and venules increased endothelial intercellular adhesion molecule-1 (ICAM-1) expression and augmented retention of LPS-untreated naïve leukocytes [1]. Thus, endothelial mechanisms by themselves may be critical in initiating LPS-induced pathophysiological responses. However, signaling that underlies LPS-induced ICAM-1 expression in lung microvessels remains unclear.

In endothelial cells, LPS acting via toll-like receptor 4 (TLR4) initiates transcriptional upregulation of proinflammatory and adhesion molecules [2], [3]. Our previous findings showed that the magnitude of the LPS-induced increase in ICAM-1 expression in microvessels was proportional to the duration post-LPS treatment, suggesting transcriptional regulation of ICAM-1. LPS-mediated induction of adhesion molecules in endothelial cells involves activation of nuclear factor-kappa B (NF-κB) [4]–[6]. Ca^2+^, in tandem with calmodulin, can activate NF-κB and downstream gene transcription in multiple cell types [7]–[9]. Recent evidence suggests that in lung endothelial cell monolayers, increase in cytosolic Ca^2+^ underlies LPS-induced inflammatory responses [6]. However, in pulmonary microvessel segments, it remains unknown whether Ca^2+^ plays a role in the induction of LPS-mediated responses.

In lung microvessels, cytosolic Ca^2+^ plays a role in the induction of proinflammatory responses elicited by both receptor-mediated and -independent agonists [10]–[12]. Receptor-mediated agonists induce release of Ca^2+^ from endoplasmic reticulum (ER) Ca^2+^ stores, via the second messenger inositol 1,4,5-trisphosphate (IP3) [9], [10], [13]. In contrast, receptor-independent agonists initiate entry of external Ca^2+^ via plasma membrane Ca^2+^ channels [10]. Release of Ca^2+^ from ER stores is associated with modulations in the amplitude and frequency of cytosolic Ca^2+^ oscillations [9]–[11], which in turn can regulate nuclear transcription factors [9], [14]–[16]. However, in lung microvessels, both the characteristic of the LPS-induced Ca^2+^ response and its role in initiating downstream signaling remain undefined.

Toward this, we determined endothelial cytosolic Ca^2+^ responses to infusions of LPS into lung microvessels using the isolated blood-perfused rat lung model. We then established the role of Ca^2+^ in the induction of downstream responses. Our data revealed that LPS initiates release of Ca^2+^ from ER stores, which then mediates the increase in endothelial ICAM-1 expression and microvascular leukocyte retention.

## Materials and Methods

### Ethics Statement

Animal use and studies were approved by the Institutional Animal Care and Use Committee of the University of Tennessee Health Science Center. Animals were housed and maintained by our Lab Animal Care Unit accredited by the Association for Assessment and Accreditation of Laboratory Animal Care. Animals were given access to feed and water *ad libitum*, and placed on a 12 h light-dark cycle. Surgery was only done on animals that were anesthetized and confirmed to be under a surgical plane of anesthesia.

### Lung Preparation

Adult male Sprague-Dawley rats weighing 250–350 g were anesthetized by intraperitoneal injection of Ketamine/Xylazine and exanguinated by cardiac puncture. The chest wall was opened and the pulmonary artery and left atrium were cannulated. Subsequently, the lungs and heart were excised *en bloc* and placed on a microscope stage. The lungs were constantly inflated at an airway pressure of 5 cmH2O and continuously pump-perfused at 14 ml/min with autologous blood warmed to 37°C. The pulmonary artery and left atrial pressures were maintained at 10 and 3 cmH2O, respectively. The lung surface was kept moist with saline throughout the experiment.

### Endothelial Cell Culture

Primary rat lung microvascular endothelial cells (RLMVEC) were purchased from VEC Tech (Rochester, NY) and cultured in medium 199 supplemented with growth factors. RLMVEC were used in passage numbers 3–5.

### Chemicals

LPS from E. coli (serotype 0111:B4), nuclear stain Hoechst-33342, Ficoll gradient Histopaque-10771, and rhodamine 6G (R6G) were from Sigma Aldrich (St. Louis, MO). Inhibitors Xestospongin C (XeC) and 2,5-Di-(t-butyl)-1,4-hydroquinone (t-BHQ) were from Calbiochem/EMD Millipore (Billerica, MA). KappaB-kinase NEMO-binding domain (IKK-NBD) peptide and 1-oleoyl;-2-acetyl-*sn*-glycerol (OAG) were from Enzo Life Sciences (Farmingdale, NY). Alexa-Fluor 488-conjugated goat anti-mouse secondary Ab and Alexa-Fluor 488-conjugated goat anti-rabbit secondary Ab were from Invitrogen/Life Technologies (Grand Island, NY). Mouse monoclonal anti-ICAM-1 antibody and anti-NF-κB p65 TRITC-conjugated Ab were from Santa Cruz Biotechnology (Santa Cruz, CA). Anti-phosphoSer536 NF-κB p65 antibody was from Cell Signaling (Danvers, MA). The Ca^2+^ indicator Fura2-AM was from Invitrogen. All other chemicals were from Sigma.

Agents and leukocytes were infused into microvessels in a calcium rich HEPES-buffered Ringer’s solution (HBS; 150 mM Na^+^, 5 mM K^+^, 1 mM Ca^2+^, 1 mM Mg^2+^, 10 mM glucose, 20 mM HEPES) with 4% dextran (40 kDa) and 1% fetal bovine serum. Ca^2+^-free HBS was prepared as similar to HBS, but without adding Ca^2+^. Prior to infusion into microvessels, 4% dextran and 0.05 mM EGTA were added to Ca^2+^-free HBS. Both HBS and Ca^2+^-free HBS were adjusted to a final pH of 7.4.

### Leukocyte Isolation

A portion of the autologous blood was set aside for leukocyte isolation as described previously [1]. In brief, leukocytes were isolated using a Ficoll gradient, washed with phosphate-buffered saline, counted with a hemocytometer, and labeled with R6G (2 µM). The final concentration was adjusted to 100000 leukocytes/ml in HBS.

### Real-time Fluorescence Microscopy

The isolated blood-perfused rat lung was positioned on a custom-made stage. A PE10 (BD Biosciences, Sparks, MD) microcatheter was introduced through the left atrial cannula and blood cell-free conditions were established in microvessels by flushing with HBS in a small portion of the lung. We infused either HBS or LPS (100 µg/ml) for 30 min into microvessels, and then washed off luminal LPS with infusion of HBS for 60 min.

Subsequently, R6G-labeled leukocytes were infused by microcathether into the LPS-treated microvessels and their transit through the vessel was imaged in real-time using a fluorescence microscope (BX61WI, Olympus America, Center Valley, PA) fitted with the appropriate interference filters (excitation 545 nm and emission >560 nm). At the end of leukocyte infusion, we flushed the vessels with HBS. We then captured images of several microvessels within the LPS-treated region.

Post-experiment, we analyzed the fluorescence images with Metamorph (Molecular devices, Sunnyvale, CA) and quantified the number of leukocytes retained in microvessels separately for all treatment groups.

### In situ Immunofluorescence of ICAM-1, Phospho p65 and NF-kB by Confocal Microscopy

Expression of ICAM-1, phosphorylation of NF-κB p65 subunit, and nuclear translocation of NF-κB p65 subunit were determined by *in situ* immunofluorescence as previously reported [1], [17]. In brief, following LPS (100 µg/ml) infusion for 30 min and subsequent HBS wash for 60 min, we fixed the microvessels with 3.7% paraformaldehyde. However for establishing phosphorylation of NF-κB p65 subunit, the microvessels were fixed immediately following 30 min of LPS infusion. For ICAM-1 and phosphorylation of NF-κB p65 determinations, we infused the primary Ab (10 µg/ml) for 30 min, followed by a brief HBS wash, and then a fluorophore-tagged secondary Ab (10 µg/ml) for 30 min. For NF-κB translocation determination, we infused the TRITC-tagged primary Ab for 30 min. After the antibody infusions, we washed off excess Ab with HBS and imaged microvessels with a confocal laser scanning imaging system (LSM-710; Zeiss, Thornwood, NY). In experiments to determine phosphorylation of NF-κB p65 and NF-κB p65 nuclear translocation, we infused the nuclear marker Hoechst-33342 (10 µg/ml).

### Inhibitor Pretreatment

To determine the role of IP3-mediated ER Ca^2+^ release, we pretreated microvessels for 10 min before LPS infusion with XeC (25 µM) or t-BHQ (10 µM) with Ca^2+^ free HBS. To inhibit NF-κB, we infused IKK-NBD peptide (100 µM) that selectively inhibits NF-κB activation, for 30 min before and during LPS infusion.

### Cytosolic Ca^2+^ Determinations in Microvessels

To determine endothelial cytosolic Ca^2+^, we loaded microvessels with the cell-permeable ratiometric Ca^2+^ indicator Fura2-AM (10 µM) for 30 min. We then obtained images of the microvessels with our fluorescence microscope by exciting sequentially at 340 and 380 nm, and collecting the fluorescence emission at 515 nm. Images were obtained at 10 s intervals. The fluorescence emissions from both 340 and 380 excitations were quantified at a single region along a vessel wall. Changes in Ca^2+^ were indicated as changes in the ratio of the fluorescence emissions at 340 and 380 nm (F340/F380).

F340/F380 oscillation amplitude in lung microvessels were determined as reported previously [11]. In brief, temporal changes in F340/F380 for venules and capillaries were plotted separately. Then, the oscillation amplitude was quantified for three consecutive oscillations and the mean was taken as the amplitude of Ca^2+^ oscillation. The results were confirmed by a blinded analysis.

To quantify baseline F340/F380 in a vessel, the value at a single region on the vessel wall was determined for 15 consecutive images, and then the mean F340/F380 quantified.

### Cytosolic Ca^2+^ Determinations in RLMVECs

RLMVECs were loaded with Fura2-AM for 40 min and washed with cell culture media. The cells were then imaged using parameters described above for microvessels. Images of cells were obtained from several distinct regions within a culture dish. After obtaining a series of images at baseline, either LPS (1 µg/ml) or OAG (100 µM) was added to the media and the imaging continued. To determine the role of IP3 receptor inhibition, RLMVECs were pretreated with XeC (10 µM) for 20 min before agonist treatment.

To quantify F340/F380 in RLMVEC, an outline was drawn around cells in the image field and the F340/F380 calculated. For each RLMVEC, F340/F380 was calculated from three consecutive images and the mean taken as the F340/F380 for that cell. For each culture dish, a minimum of three cells per region and greater than three regions per dish were analyzed.

### Statistics

All data are reported as mean±SEM. All multiple groups were compared with Kruskal-Wallis One Way ANOVA on Ranks followed by pair-wise multiple comparisons by Dunn’s method.

## Results

### LPS Increased the Magnitude of Cytosolic-Ca^2+^ Oscillation in Lung Microvessels

**Figure 1:**
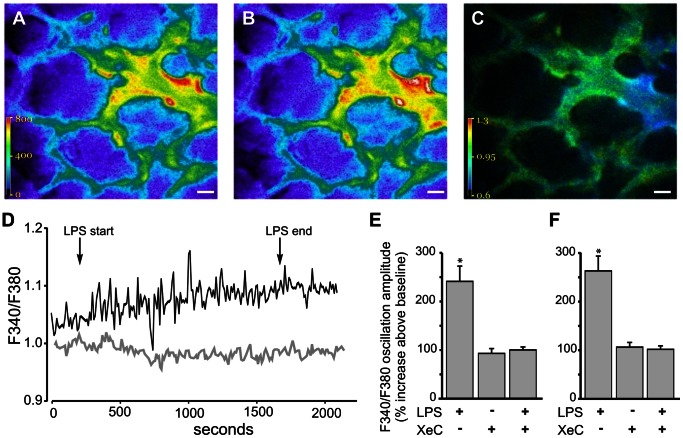
Ca^2+^ response in lung microvessels. **A–C** Pseudocolor images show fluorescence emission in response to exciting Fura2-AM loaded microvessels at 340 nm (A) and 380 nm (B). The corresponding 340/380 fluorescence ratio was quantified on a pixel-by-pixel basis and displayed as a pseudocolored image (C). Scale bar = 20 µm. **D**. Tracings show temporal changes in F340/F380 in response to LPS infusions (100 µg/ml) into control (black tracing) and XeC-pretreated (gray tracing) lung microvessels. **E, F** Bar graphs show composite increase in the LPS-induced F340/F380 oscillation amplitude above baseline values for venules (E) and capillaries (F). Mean±SE. n = 4 vessels from 3 lungs per group. *-p<0.05 compared to XeC and XeC+LPS treatment groups.

Fluorescence images of lung microvessels, loaded with the cytosolic Ca^2+^ indicator Fura2-AM (**Figure 1A, B**) were captured at 10 s interval and the corresponding F340/F380 fluorescence ratio images (**Figure 1C**) were derived in real-time for the entire duration of an experiment. At baseline, mean F340/F380 in venules was slightly higher than that in capillaries (2.8±1.6%; range 0 to 9%; n = 6 lungs). Temporal changes in single vessel F340/F380 quantified from the images indicated that infusion of LPS into microvessels, induced a rapid increase in the amplitude of Ca^2+^ oscillations (**Figure 1D**). XeC infusion into microvessels, prior to LPS infusion, blunted the LPS-induced response (Figure 1D). As XeC blocks IP3 receptors on the ER, the inhibition suggested that LPS induced an IP3-mediated Ca^2+^ release from the ER. LPS induced a more than 2-fold increase in oscillation amplitude in both venules and capillaries (**Figure 1E, F**). XeC was effective in inhibiting the response in both microvessel segments (**Figure 1E**, F).

**Figure 2:**
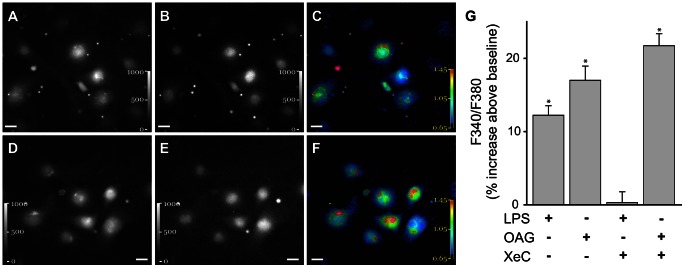
Ca^2+^ response in RLMVEC. **A–F** Images show fluorescence emission intensity in response to exciting Fura2-AM loaded RLMVEC at 340 nm (A, D) and 380 nm (B, E) The corresponding 340/380 fluorescence ratio was quantified on a pixel-by-pixel basis and displayed as pseudocolored images (C, F). The images were obtained at baseline (A–C) and after OAG (100 µM) treatment (D-F). Scale bar = 20 µm. **G**. Bar graph shows increase in F340/F380 above baseline in RLMVEC treated with LPS (1 µg/ml) or OAG (100 µM), with and without XeC (10 µM) pretreatment (20 min). Mean±SE. n = 2−3 separate experiments and >50 RLMVECs per treatment group. *-p<0.05 compared to XeC+LPS treatment group.

To establish that XeC did not block entry of external Ca^2+^ via plasma membrane Ca^2+^ channels, we determined Ca^2+^ responses in RLMVEC. Fura2-AM loaded RLMVEC were treated with the OAG, a cell-permeable diacylglycerol analog, which induces entry of external Ca^2+^ through transient receptor potential canonical (TRPC) 6 on the plasma membrane [6], [18]. OAG increased F340/F380 above baseline levels in RLMVEC (**Figure 2A–F**). Pretreating RLMVECs with XeC, did not affect the OAG-induced increase in cytosolic Ca^2+^ (**Figure 2G**). In contrast, as observed in microvessels, XeC blunted the LPS-induced increase in cytosolic Ca^2+^ increases in RLMVEC (**Figure 2G**). Thus, external Ca^2+^ entry via plasma membrane Ca^2+^ channels remained unaffected by XeC.

### ER Ca^2+^ Release Mediated LPS-induced ICAM-1 Expression in Lung Microvessels

**Figure 3:**
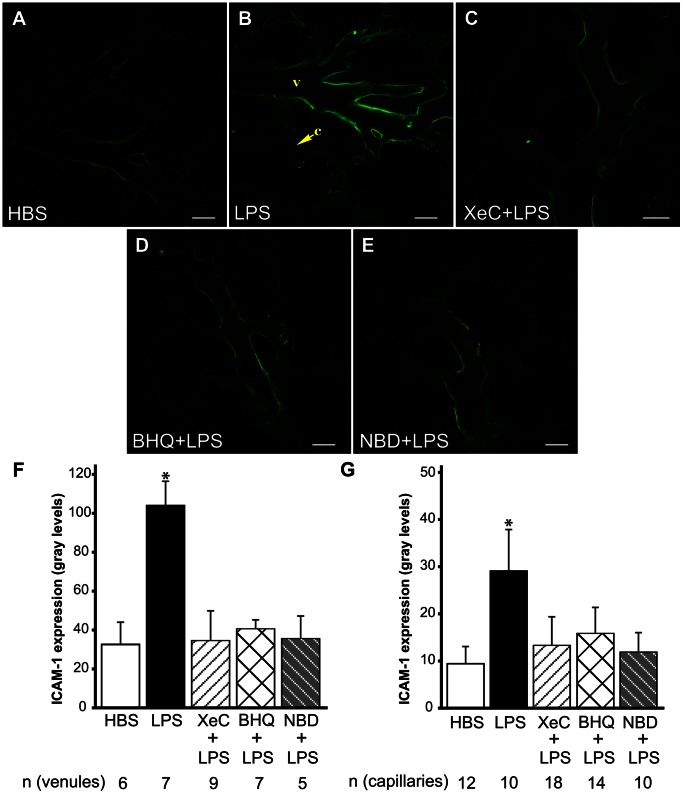
ICAM-1 expression in lung microvessels. **A–E** Confocal images show immunofluorescence of ICAM-1 in lung microvessels following treatments as indicated. Treatment durations were as outlined in *Methods*. *v*-venule, *c*-capillary. Scale bar = 20 µm. **F, G** Bar graphs show ICAM-1 immunofluorescence intensity quantified along the vessel wall over the length of single microvessels for both venules (F) and capillaries (G). Mean±SE. *n* - number of vessels analyzed per group from a minimum of 2 lungs per group, except for XeC+LPS (3 lungs) and NBD (1 lung) groups. *-p<0.05 compared to HBS, XeC+LPS and NBD+LPS groups. *BHQ* = t-BHQ, *NBD* = IKK-NBD.

We previously established that LPS increased ICAM-1 expression in pulmonary capillaries and venules [1]. Here we defined the role of cytosolic Ca^2+^ in this response. *In situ* immunofluorescence images of ICAM-1 indicated that as compared to HBS, LPS infusion into microvessels increased venular and capillary ICAM-1 expression (**Figure 3A,B**). To define whether ER Ca^2+^ played a role in this response, we determined ICAM-1 expression in the presence of inhibitors of ER store Ca^2+^ release. In microvessels pretreated with XeC, the LPS-induced increase in ICAM-1 expression was lower (**Figure 3C**). In addition, pretreating microvessels with t-BHQ, which inhibits the Ca^2+^-ATPase pumps on ER [15], also limited the LPS-induced ICAM-1 expression (**Figure 3D**). XeC- and t-BHQ-dependent inhibition was evident in both venules and capillary microvessel segments (**Figure 3F, G**). Taken together, the data suggest a role for ER Ca^2+^ in the induction of LPS-induced ICAM-1 response in lung microvessels.

As NF-κB is known to play a role in the induction of LPS-induced responses, we blocked NF-κB activation with the peptide inhibitor, IKK-NBD. Infusion of IKK-NBD into microvessels prior to and during LPS infusion, blunted the LPS-induced increase in ICAM-1 expression (**Figure 3E**). The inhibition was evident in both venular and capillary microvessel segments (**Figure 3F, G**).

### LPS-induced Leukocyte Retention in Lung Microvessels was Dependent on ER Ca^2+^ Release

**Figure 4:**
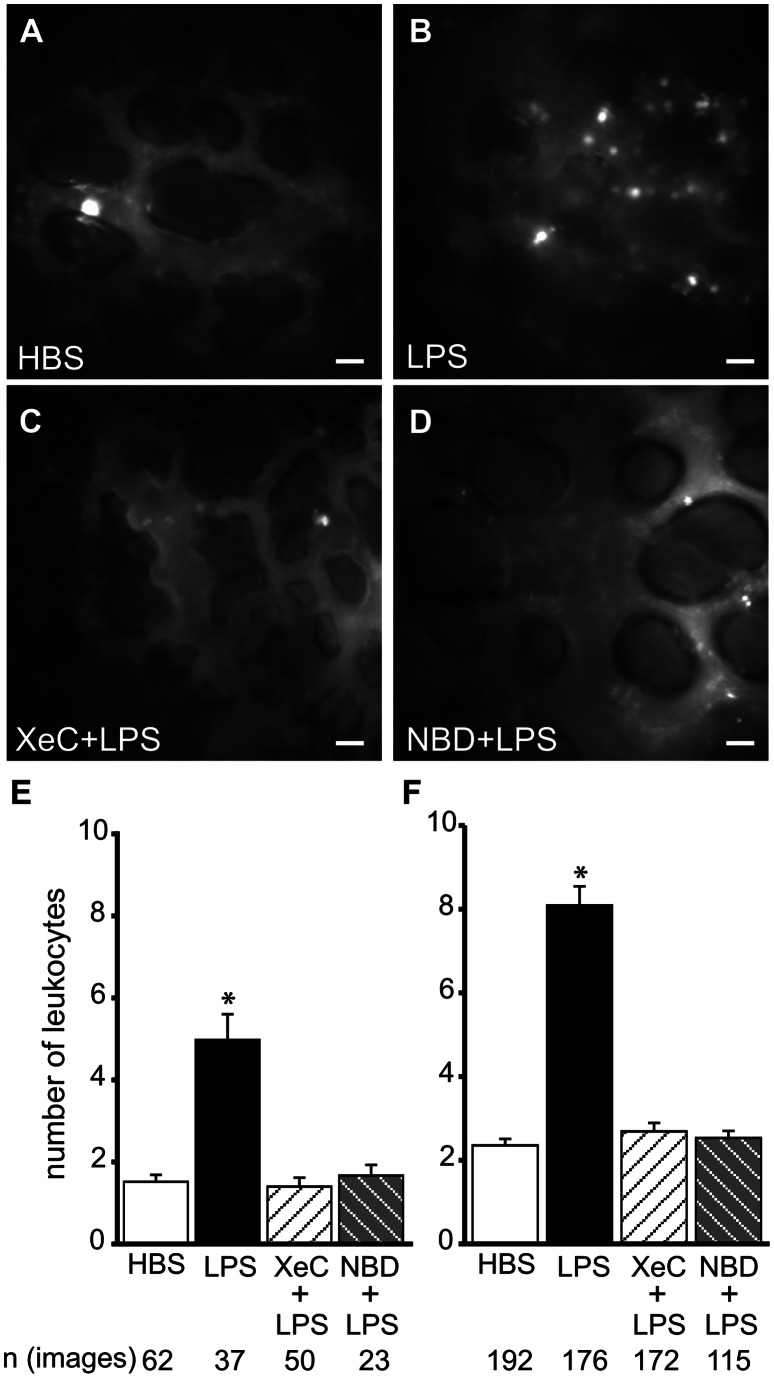
Leukocyte retention in lung microvessels. **A–D** Fluorescence images show retention of R6G-labeled leukocytes in lung microvessels following treatments, as indicated. Treatment durations and agent concentrations were as outlined in *Methods*. Scale bar = 20 µm. **E, F** Bar graphs show number of retained leukocytes per image frame in venules (E) and capillaries (F) following the indicated treatments. Mean±SE. *n* - number of images analyzed per group (3 lungs per group). *-p<0.05 compared to HBS, XeC+LPS and NBD+LPS groups. *NBD* = IKK-NBD.

Since LPS increased leukocyte retention in lung microvessel segments as reported previously [1], we determined whether this response was mediated by ER Ca^2+^ release. Fluorescence images reveal that retention of R6G-labeled naïve leukocytes in LPS-treated microvessels was higher than in HBS-treated leukocytes (**Figure 4A, B**). To define the role of ER Ca^2+^ in leukocyte retention, we first infused XeC into microvessels. Then, we infused LPS into these microvessels followed by infusion of R6G-labeled leukocytes. As evident from the fluorescence images, XeC completely blocked the increase in LPS-induced leukocyte retention (**Figure 4C**). This inhibition was significant in both capillaries and venules (**Figure 4E, F**). These data suggest that LPS-induced leukocyte retention in lung microvessels was dependent on ER Ca^2+^ release. In addition, blocking NF-κB activation by infusing IKK-NBD peptide into microvessels prior to LPS infusion, also blunted leukocyte retention (**Figure 4D–F**).

### ER Ca^2+^ Release Mediated LPS-induced NF-κB Activation in Lung Microvessels

**Figure 5:**
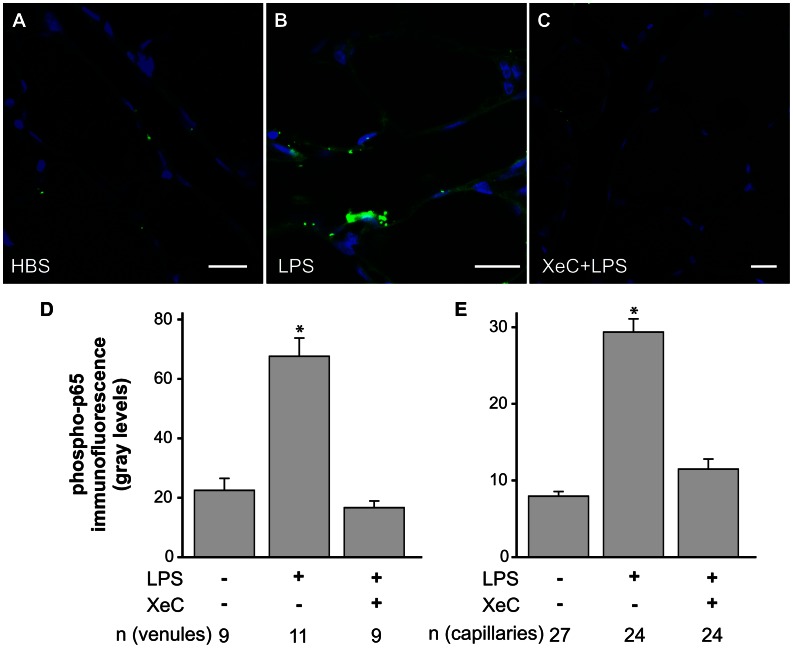
Phosphorylation of p65. **A–C** Confocal immunofluorescence images show phosphorylation of the NF-κB p65 subunit in lung microvessels (green) and fluorescence of the nuclear marker Hoechst-33342 (blue) for the indicated treatments. Treatment durations and agent concentrations were as outlined in *Methods*. Scale bar = 20 µm. **D, E** Bar graphs show NF-κB p65 phosphorylation levels along the vessel wall over the length of single microvessels for both venules (D) and capillaries (E). Mean±SE. *n* - number of vessels analyzed per group. Each treatment repeated in 3 lungs each. *-p<0.05 compared to HBS and XeC+LPS groups.

To establish whether LPS activates NF-κB in an ER Ca^2+^-dependent manner, we determined phosphorylation of the p65 subunit of NF-κB in lung microvessels by *in situ* immunofluorescence. Confocal immunofluorescence images revealed that LPS increased p65 phosphorylation (**Figure 5** **A, B**). The images also indicated that pretreating microvessels with XeC blunted the LPS-induced p65 phosphorylation (**Figure 5C**). The XeC-dependent inhibition was evident in both venules and capillaries (**Figure 5D, E**). Together, the data suggest that LPS-induced ER Ca^2+^ release mediated the phosphorylation of NF-κB p65 subunit in lung microvessels.

**Figure 6:**
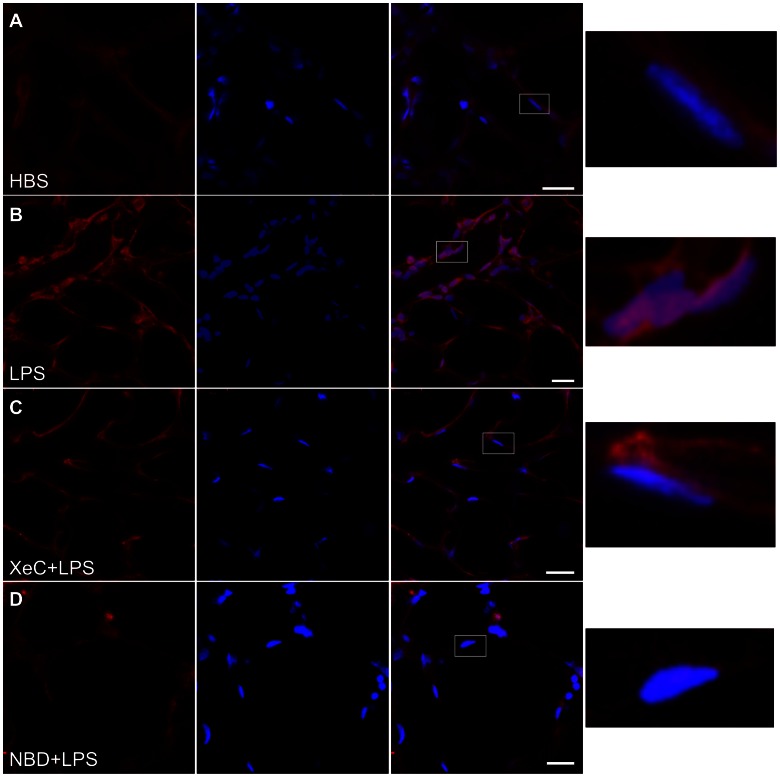
Nuclear localization of NF-kB. Confocal images show immunofluorescence of NF-κB p65 subunit in lung microvessels (red, left column), fluorescence of the nuclear marker Hoechst-33342 (blue, column 2) and merge (column 3) for the indicated treatments. Pink color in the merged images indicates colocalization of NF-κB immunofluorescence and nuclear marker fluorescence. Treatment durations were as outlined in *Methods*. Nuclear localization of NF-κB immunofluorescence in the region marked by the rectangles (column 3 images) is shown magnified on far right column. Again, pink color indicates localization of NF-κB immunofluorescence (red) and nuclear marker fluorescence (blue). Treatment groups repeated in 3 lungs, except for NBD treatment group (n = 1). Note the higher colocalization in LPS-treated microvessels (B), compared to HBS-treated (A), and XeC- (C) and NBD-pretreated microvessels. *NBD* = IKK-NBD.

Since the p65 subunit of NF-κB translocates to the nucleus upon activation [19], [20], we determined LPS-induced nuclear localization of NF-κB p65 by *in situ* immunofluorescence imaging. As evident from the confocal images, overlap between the NF-κB immunofluorescence and nuclear fluorescence was greater in LPS-treated microvessels compared to HBS-treated vessels (**Figure 6A, B**). Pretreating microvessels with XeC blunted the LPS-induced translocation, indicating that NF-κB activation and its translocation to the nuclear region were dependent on ER Ca^2+^ release (**Figure 6C**). In addition, the translocation was also lower in microvessels pretreated with the IKK-NBD peptide (**Figure 6D**).

## Discussion

We show here for the first time that infusion of LPS into lung microvessels, induced release of Ca^2+^ from ER stores and attendant increase in the amplitude of cytosolic Ca^2+^ oscillations. In addition, our data also reveal that store Ca^2+^ release mediated the nuclear translocation of NF-κB. Thus, the current findings clearly attribute a major role for Ca^2+^ release from endothelial ER stores in the induction of LPS-induced inflammatory responses in lung microvessels.

LPS infusion into microvessels induced both the phosphorylation and nuclear translocation of the p65 subunit of NF-κB. However, pretreating microvessels with XeC limited both the phosphorylation and translocation, suggesting that Ca^2+^ release from ER stores mediated these responses. Further, the cell-permeable IKK-NBD peptide also blocked the translocation. IKK-NBD blocks association of the NF-κB essential modifier with the IKK complex and thus, NF-κB activation [21]. Together the data suggest that LPS-mediated released of ER Ca^2+^ caused activation of NF-κB, leading to its nuclear translocation. While LPS-mediated NF-κB activation is well established [5], [22], [23], a role for store Ca^2+^ in this process is less defined. In addition, while store Ca^2+^ release has been indicated to mediate LPS responses in inflammatory cells [19], [24], [25], such a role remains unknown in endothelial cells. Our studies provide the first indication that release of Ca^2+^ from ER stores may play a role in LPS-induced NF-κB activation. Calmodulin, a calcium sensor that is activated upon binding Ca^2+^, may be an intermediary in the Ca^2+^-dependent NF-κB activation [7], [22], [26], [27]. Whether calmodulin mediates the responses in pulmonary vasculature remains to be established. Since, even small increases in Ca^2+^ are reported to elicit NF-κB-dependent responses [7], [16], [22], it is possible that the present responses observed in the pulmonary microvessels may be sufficient to initiate activation of NF-κB and attendant downstream signaling.

Studies on LPS-induced responses, implicate influx of external Ca^2+^ via TRPC-permeable channels as the primary mechanism underlying cytosolic Ca^2+^ increase and downstream signaling [6], [28]–[30]. However, in this study the increase in Ca^2+^ oscillations were mediated by store Ca^2+^ release, as evinced by the inhibition of the LPS-induced response by the ER IP3 receptor blocker, XeC. This interpretation is supported by the finding that in RLMVEC, XeC did not inhibit cytosolic Ca^2+^ increases by OAG. As OAG induces entry of external Ca^2+^ via TRPC6 channels [6], [18], it is evident that XeC pretreatment had no direct effect on plasma membrane Ca^2+^ channels. In addition, using fluorescent probes that report store Ca^2+^ levels it has been shown previously that XeC inhibits store Ca^2+^ release, but not entry of external Ca^2+^ in lung microvessels [10]. Taken together, our data clearly suggests that in lung microvessels the primary mechanism of endothelial Ca^2+^ increase by LPS is via IP3-dependent release of ER store Ca^2+^.

Previous studies in lung microvessels suggest that receptor-mediated agonists initiate IP3-dependent release of store Ca^2+^ [10]. Since, the LPS-induced responses are also receptor-mediated, it follows that in lung microvessels LPS is likely to initiate Ca^2+^ release from ER stores. This possibility is supported by the observation that inhibiting ER Ca^2+^-ATPase pumps also blocked the LPS-induced ICAM-1 release. The difference in Ca^2+^ responses observed in this study from that recently reported in lung endothelial cells [6] may be attributed to signaling differences between micro- and macro-vascular endothelial cells. It is well established that agonist-induced responses and the underlying signaling are different in macro- vs micro-vascular endothelium [31]. Our findings determined via focal infusions of LPS reveal signaling specific to microvessels and thus, the microvascular endothelium. These interpretations are further supported by the inhibition of LPS-induced Ca^2+^ increase in primary RLMVEC by blocking ER store Ca^2+^ release. Thus in pulmonary microvessels, we interpret that LPS signals via release of Ca^2+^ from ER stores.

The effect of LPS on lung microvessels was to increase the amplitude of cytosolic Ca^2+^ oscillations. Modulations in frequency and amplitude of cytosolic Ca^2+^ oscillations underlie several Ca^2+^-dependent signaling pathways in various cell types [4], [5], [9], [14]–[16], [32]–[34]. In lung microvessels, an increase in the amplitude of cytosolic Ca^2+^ oscillation augmented reactive oxygen species production and P-selectin expression [11]. Further, changes in Ca^2+^ oscillations activate transcriptional events, possibly via the action of calcium/calmodulin kinases [14]–[16]. These reports support our finding that increases in the amplitude of endothelial cytosolic Ca^2+^ oscillations induced by LPS lead to NF-κB translocation to the nucleus and an increase in ICAM-1 expression.

In the present study, ICAM-1 expression was elevated at 90 minutes post-LPS infusion. However, we reported that ICAM-1 expression was detectable in both venules and capillaries as early as 30 minutes after initiating LPS infusion [1]. In contrast, in umbilical vein and pulmonary artery endothelial cells, induction of ICAM-1 expression by inflammatory stimuli, including LPS, thrombin, tumor necrosis factor-α, and interleukin-1β, occurs several hours after stimulus application [35]–[38]. This difference suggests that transcription of ICAM-1 is rapid in microvessels. LPS and other proinflammatory stimuli induce transcription in two stages; a first stage occurring within 30 minutes involves induction of immediate-early response genes, and a second stage occurring between 1 and 4 hours leads to the induction of primary and secondary response genes [7], [39]–[43]. In LPS-treated cells, these two stages parallel the two waves of NF-κB recruitment to the nucleus [44]. As ICAM-1 expression was evident in lung microvessels after only 30 min of LPS treatment, it is likely ICAM-1 induction is part of the early response. It has been posited that factors involved in transcriptional regulation are primarily upregulated during the rapid first stage [42]. Interestingly, antibody ligation of ICAM-1 in endothelial cells, a procedure that mimics leukocyte adhesion [45], itself initiates transcription of an early response gene, c-Fos [46] and the transcription factor complex, activator protein 1 [47]. Moreover, we showed that leukocyte retention in lung microvessels parallels the increase in endothelial ICAM-1 expression, suggesting a role for ICAM-1 in the transcription process [1]. Taking these together, we speculate that in lung microvessels LPS initiates rapid induction of ICAM-1 and concomitant increase in leukocyte adhesion to endothelium. Leukocyte adhesion then leads to further rapid transcription in the endothelium. Thus, the rapid increase in ICAM-1 expression may facilitate fast microvascular responses to an inflammatory insult. However, additional studies are required to elucidate this possibility in lung microvessels.

We reported that LPS-induced ICAM-1 expression in venules was higher than that in capillaries [1]. Data from the present study and other reports on endothelial cells indicate that induction of ICAM-1 expression is mediated by Ca^2+^ [6], [48]. Thus, it is possible that differences in Ca^2+^ signaling underlie differences in ICAM-1 expression in venules and capillaries. While the increase in the amplitude of Ca^2+^ oscillation was similar in both microvascular segments, the baseline Ca^2+^ was lower for capillaries compared to venules. Since, heterogeneity in baseline Ca^2+^ can lead to differences in the induction of Ca^2+^-dependent signaling [49], it is likely that differences in venular versus capillary Ca^2+^ at baseline contributed to their dissimilar ICAM-1 expression. Whether small differences in Ca^2+^ can significantly modulate endothelial responses and the specifics of the signaling mechanisms require further consideration.

Our findings show that LPS induces IP3-dependent ER Ca^2+^ release and downstream responses in lung microvessels. Inhibiting IP3 receptors with XeC blocked induction of ICAM-1 and leukocyte retention, suggesting that blunting ER Ca^2+^ release may limit inflammatory responses in lung microvessels. In addition, reports indicate that inhibiting IP3-dependent store Ca^2+^ release also blunts LPS-induced cytokine production in macrophages [30], [50], [51]. Leukocyte retention in lung microvessels followed by their subsequent migration into the airspace, and cytokine production by activated macrophages are major characteristics of LPS-induced lung inflammation and injury. Mitigating these LPS-induced responses attenuates injury and improves survival [52]–[54]. Thus, limiting release of Ca^2+^ from ER stores by blocking IP3 receptors may be effective in blunting LPS-induced inflammation and injury, and thus, facilitate survival.

Thus, our data obtained using real-time fluorescence imaging reveal a new signaling pathway for LPS-induced responses in pulmonary microvessels that involves IP3-mediated Ca^2+^ release leading to increase in cytosolic Ca^2+^ oscillations, activation followed by nuclear translocation of NF-κB, an increase in ICAM-1 expression and attendant leukocyte retention. These findings may lead to development of novel intervention strategies for endotoxin-induced lung injury.
